# Efficacy of Early Postoperative Subthreshold Micropulse Laser Therapy in Preventing Persistent Macular Oedema in Patients After Epiretinal Membrane Surgery

**DOI:** 10.3390/biomedicines13092113

**Published:** 2025-08-29

**Authors:** Alicja Ziontkowska-Wrzałek, Monika Dzięciołowska, Krzysztof Safranow, Anna Machalińska

**Affiliations:** 1 First Department of Ophthalmology, Pomeranian Medical University, Al. Powstańców Wielkopolskich 72, 70-111 Szczecin, Poland; aziontkowskawrzalek@gmail.com (A.Z.-W.); monika_md@poczta.onet.pl (M.D.); 2 Department of Biochemistry and Medical Chemistry, Pomeranian Medical University, Al. Powstańców Wielkopolskich 72, 70-111 Szczecin, Poland; chrissaf@mp.pl

**Keywords:** epiretinal membrane, ERM, subthreshold micropulse laser therapy, SMLT, persistent macular oedema, optical coherence tomography, OCT, OCT angiography, multifocal electroretinography, microperimetry

## Abstract

**Background/Objectives:** Epiretinal membrane (ERM) is often associated with macular thickening and foveal intraretinal fluid. The aim of this study was to evaluate the efficacy of early postoperative SMLT (577 nm) in preventing persistent macular oedema and to assess its impact on selected functional and morphometric retinal parameters after ERM peeling. **Methods:** A total of 68 pseudophakic patients with ERMs were enrolled and randomly assigned (1:1) to a laser group or a nonlaser control group. SMLT was performed one month after PPV. The functional and morphometric retinal parameters were assessed preoperatively and at one and four months postoperatively via optical coherence tomography (OCT), OCT angiography (OCTA), multifocal electroretinography (mfERG), and microperimetry. **Results:** The reduction in total retinal volume between the first and fourth postoperative months was significantly greater in the SMLT group than in the control group (*p* = 0.02). No significant differences in functional parameters were found between the groups. A more substantial reduction in total retinal volume post-SMLT was associated with greater baseline macular thickness, a more advanced ERM stage, worse baseline visual acuity, greater fixation stability, lower initial macular sensitivity and lower preoperative *p*-wave amplitude in ring R1 on mfERG. **Conclusions:** SMLT may be considered a therapeutic option in patients with advanced ERM stages and low preoperative visual acuity.

## 1. Introduction

The epiretinal membrane (ERM) is a prevalent retinal condition, with studies indicating that the occurrence rate ranges from 7% to 11.8% in the general population, with this incidence increasing with age. The primary treatment for symptomatic ERMs is pars plana vitrectomy (PPV) with membrane peeling, as no pharmacological therapies have been proven effective [1]. The presence of an ERM is commonly associated with macular thickening [2] and intraretinal fluid accumulation in the foveal region [1,3].

Surgical removal of ERMs, often combined with internal limiting membrane (ILM) peeling, typically results in progressive improvements in retinal morphology and visual function [4,5]. The restoration of retinal architecture, including the resolution of macular thickening and intraretinal fluid, may require up to 24 months following surgery [6].

However, in a subset of patients, persistent macular oedema may remain [7], leading to severe consequences [8], including irreversible tissue damage and retinal cell death, ultimately resulting in permanent visual impairment [9]. Its pathophysiological mechanism is associated primarily with several factors, including inflammatory processes, the mechanical traction exerted on the retina before and during membrane peeling, and the disruption of the blood‒retina barrier due to intraocular manipulation, which stimulates the release of growth factors and inflammatory cytokines [7]. Among the proposed mechanisms, traction stress remains the primary cause of macular oedema in ERMs [2]. This form of oedema typically presents with minimal or no angiographic leakage and only a mild inflammatory response, which distinguishes it from vascular or inflammation-driven forms [2,10,11].

In the absence of effective methods to further improve anatomical and functional outcomes after ERM peeling, subthreshold micropulse laser therapy (SMLT) represents a promising therapeutic option.

SMLT delivers repeated, short-duration laser pulses separated by cooling intervals to avoid thermal damage. Unlike conventional photocoagulation, SMLT does not produce visible retinal burns and leaves no structural traces detectable by imaging techniques such as optical coherence tomography (OCT) or fundus autofluorescence. Its therapeutic effect is based on sublethal stimulation of the retinal pigment epithelium (RPE), leading to the upregulation of heat shock proteins (HSPs), which act as cellular “rescue” signals that normalize RPE function and modulate cytokine and chemokine expression. This low-intensity, high-density approach promotes neuroprotection, reduces chronic inflammation, improves mitochondrial function, and downregulates vascular endothelial growth factor (VEGF) expression in Müller cells. Additionally, SMLT has been shown to inhibit microglial activation and support retinal tissue homeostasis through localized, reparative immune responses [2,11,12].

SMLT has been applied in a wide range of retinal pathologies, including non-surgical indications such as diabetic macular oedema (DME), central serous chorioretinopathy (CSCR), proliferative diabetic retinopathy (PDR), nonexudative age-related macular degeneration (AMD), and macular oedema secondary to branch retinal vein occlusion (BRVO) [12,13,14,15,16], as well as post-surgical macular oedema following cataract or retinal detachment surgery [17,18,19].

This study evaluated the impact of SMLT on both anatomical and functional retinal outcomes in patients after surgical peeling of the ERM, with a particular focus on its early postoperative application to prevent persistent macular oedema. The functional assessment included both electrophysiological testing and microperimetry. To the best of our knowledge, this is only the second prospective study to investigate the effects of early postoperative SMLT following ERM removal, and the first to incorporate advanced functional assessments—specifically multifocal electroretinography and microperimetry—in conjunction with OCT-based structural analysis. By integrating these complementary evaluation methods within a standardized treatment protocol and follow-up period, our study provides a more comprehensive understanding of macular recovery than has been offered in previous research.

## 2. Materials and Methods

### 2.1. Study Group

This prospective, randomized, controlled, interventional, single-centre study included 68 patients who were scheduled to undergo surgical removal of an ERM. Eligible participants were diagnosed with a visually significant ERM either associated with metamorphopsia or causing a marked reduction in best-corrected visual acuity and were pseudophakic at the time of enrolment. Patients with coexisting ocular or retinal diseases other than ERMs or with clinical signs of active or recent intraocular inflammation were excluded from the study. The participants were classified preoperatively according to the Govetto staging system based on OCT findings [20] and were randomly assigned at a 1:1 ratio to either the laser treatment group or the control (nonlaser) group. All patients underwent standard 25-gauge three-port PPV under peribulbar anesthesia (2% lignocaine and 0.5% bupivacaine). Vitrectomy was performed with intravitreal triamcinolone acetonide assistance to aid visualisation of the posterior hyaloid and facilitate posterior vitreous detachment when necessary. ERM was completely removed using retinal forceps (Maculorhexis Forceps 25G, FCI, Paris, France), with peeling of the ILM after trypan blue staining, employing the pinch-and-peel technique. Peripheral vitrectomy was completed, followed by fluid–air exchange and subsequent air–gas exchange with sulfur hexafluoride (Arceole pure SF_6_, ARCAD Ophta, Toulouse, France), selected to provide temporary internal tamponade and promote retinal adherence while minimizing the duration of gas persistence. All surgical procedures were performed by the same experienced vitreoretinal surgeon (A.M.). Follow-up examinations were conducted at three time points: before surgery, one month after surgery, and four months after surgery. SMLT was performed one month after PPV surgery.

### 2.2. Ophthalmological Questionnaire

All participants completed a standardized ophthalmological questionnaire prior to undergoing PPV. The questionnaire included the following items: (1) the presence of metamorphopsia at the time of evaluation (response options: 1–Yes, 0–No); and (2) the estimated duration of metamorphopsia symptoms, categorized as: 1—less than 6 months, 2—6 to 12 months, or 3—more than 12 months.

### 2.3. Ophthalmic Examinations

The examinations included measurements of best-corrected visual acuity (BCVA) via both Snellen and ETDRS charts, as well as retinal imaging with enhanced depth imaging optical coherence tomography (Spectralis EDI-OCT, Heidelberg Engineering, Heidelberg, Germany) and OCT angiography (OCTA) of the macula (Heidelberg Engineering, Heidelberg, Germany). BCVA was measured twice, and the better of the two values was recorded. All measurements were performed by the same examiner under identical testing conditions to ensure consistency.

The following parameters were assessed: retinal thickness in the nine Early Treatment Diabetic Retinopathy Study (ETDRS) subfields (μm), total retinal volume (mm^3^), subfoveal choroidal thickness (µm), and choroidal area (mm^2^). Additionally, OCTA analysis included measurement of the foveal avascular zone (FAZ) area in both the superficial (SVC) and deep vascular complexes (DVC), expressed in mm^2^. For OCT-based parameters (retinal thickness, total retinal volume), image acquisition was repeated as many times as necessary to obtain high-quality scans according to the device’s quality index; the final values were automatically calculated by the Heidelberg OCT system. Manual image analysis included measurements of the FAZ area in SVC and DVC, subfoveal choroidal thickness, and total choroidal area, all of which were measured once by a single experienced examiner, with the accuracy verified by the study supervisor.

Wide-field fundus imaging was conducted via the Optos device (Optos PLC, Dunfermline, Scotland).

All ophthalmic examinations were conducted by a single experienced examiner to minimize inter-observer variability, and data extraction was carried out by a second examiner.

### 2.4. Multifocal Electroretinography (RetiScan System, Roland Consult, Germany)

The preparation for the mfERG involved maximal pupil dilation via 10% phenylephrine hydrochloride and spectacle correction adjusted for a 0.3-metre distance. Unilateral stimulation was conducted using a black and white matrix of 103 hexagons, with a distortion factor set at 4. Importantly, recordings were obtained according to the International Society for Clinical Electrophysiology of Vision (ISCEV) standards [21]. Central fixation was applied. The matrix covered 30° of the centre to edge from the displayed fixation point. The stimulus luminance was 100 cd/m^2^, with a Michelson contrast of 97%. A thread DTL electrode (Diagnosys LLC, Lowell, MA, USA) was used as the active electrode. A gold disc skin electrode (Roland Consult, Brandenburg, Germany) placed at the ipsilateral outer canthus served as the reference electrode, and a similar electrode attached in the middle of the forehead (Fpz) served as the ground. To minimize random bias, each eye was tested six times, and the results were averaged. Additionally, automatic double postacquisition smoothing and line interference reduction were applied, along with manual correction of cursor positioning. The recording system was set to the following parameters: amplifier sensitivity of 20 μV/div; filters set to 10–300 Hz; notch filters turned off; plot time of 83 ms; and an artefact reject threshold of 8% (for amplifier range ±100 μV), according to local laboratory standards. The response density (nV/degree^2^) and P1-wave implicit time (ms) were analysed in six concentric rings.

All mfERG examinations were performed by the same trained orthoptist to ensure consistency. Manual correction of cursor positioning and analysis of the results were conducted by a trained ophthalmologist.

### 2.5. Microperimetry (Macular Integrity Assessment, MAIA; CenterVue, Italy)

Microperimetry was performed via the MAIA microperimeter in the “New Expert Exam” mode to assess macular threshold sensitivity and fixation stability. The examination employed the 4-2 (full threshold) strategy. The average test duration was approximately 4–7 min per eye.

A standard 10° (10° area, pattern of 3 rings, 37 points) grid was used for testing. This grid samples the central 10° of the macula and predominantly covers the foveal region (within a 2.5° radius) and the inner parafovea (between a 2.5° and 4° radius), with loci distributed concentrically at 0°, 1°, 2°, and 3° eccentricities from the fovea.

Stimuli were presented as Goldmann III achromatic spots against a dim white background of 4 apostilbs (asb). The stimulus duration was 200 ms, and the stimulus dynamic range covered 0–36 dB, with the maximum luminance reaching 1000 asb. Real-time eye tracking was performed at a rate of 25 Hz to ensure accurate fixation monitoring.

The following absolute values were recorded: (1) average threshold (dB), defined as the mean retinal sensitivity across all tested loci; (2) fixation stability, reported as (I) P1—the percentage of fixation points within a 2° diameter circle and (II) P2—the percentage within a 4° diameter circle; and (3) fixation variability (BCEA), assessed via the bivariate contour ellipse area at both 63% and 95% confidence levels, which represents the proportion of fixation points enclosed within each ellipse. Each ellipse was characterized by (i) horizontal and vertical semiaxes (in degrees), (ii) area (in square degrees), and (iii) orientation of the major axis relative to the horizontal axis (measured counterclockwise). All tests were administered by the same trained orthoptist, ensuring procedural consistency. Fixation stability evaluation, as well as manual review and interpretation of the results, was performed by a trained ophthalmologist. Microperimetry measurements were repeated as needed to achieve reliable results without fixation losses.

### 2.6. Subthreshold Micropulse Laser Therapy

SMLT was delivered via a 577 nm solid-state diode yellow laser (Supra 577; Quantel Medical, Clermont-Ferrand, France). SMLT was applied to the macular area via thirty 5 × 5 spot sizes of 160 µm with zero-spot spacing, including the fovea. A contact lens with a laser magnification of 1.06× Area Centralis^®^ (Volk Optical, Mentor, OH, USA) was used. The threshold power was determined by setting the power to a fixed low value 250 mW. The exposure duration was 200 ms, and the laser was switched to micropulse mode at a duty cycle of 5%. Parameters were selected based on previously published protocols [22,23] and applied in a panmacular, dense treatment mode to ensure homogeneous energy delivery to the targeted tissue. All patients were treated by the same ophthalmologist (M.D.).

### 2.7. Statistical Analysis

The normality of distributions was determined via the Shapiro–Wilk test. As most continuous variables deviated significantly from normal distribution, nonparametric tests were applied. The Mann–Whitney U test was used for comparisons of continuous or ordinal variables between groups. Spearman’s rank correlation coefficient (Rs) was used to assess the strength of associations between continuous or ordinal variables. Two-tailed Fisher’s exact test was used to compare categorical variables. Quantitative data are presented as medians with interquartile ranges (IQRs) while number and percentage are reported for qualitative data. A *p*-value of less than 0.05 was considered statistically significant. The statistical analysis was conducted via Statistica 13 software.

## 3. Results

### 3.1. Preoperative Characteristics of the Study Population

Sixty-eight patients with ERMs who underwent PPV with ERM and ILM peeling were included in the study. Patients were classified according to the Govetto stages and randomly assigned at a 1:1 ratio to a subthreshold micropulse laser group or a nonlaser control group. The preoperative clinical characteristics of the patients in the SMLT and control groups are summarized in Table 1. The SMLT and control groups were matched for age and sex. Moreover, no significant differences in best corrected visual acuity (BCVA), Govetto ERM stage, or the presence or duration of metamorphopsia were noted between the groups. Accordingly, no significant differences in baseline morphological or functional characteristics were found between the groups.

### 3.2. Postoperative Changes in Morphological and Functional Characteristics

To estimate the restorative effects of SMLT on retinal morphology and function, we performed a quantitative analysis of retinal morphology and function before and after SMLT. A complete ophthalmologic examination, OCT, OCT angiography (OCTA), microperimetry and electrophysiological testing were performed 1 day before and 3 months after SMLT. Table 2 shows the values of the postoperative parameters measured 1 month post-ERM surgery but before laser application for the two groups of eyes. No significant differences in either morphological or functional characteristics were noted between the SMLT and observation groups.

**Figure 1:**
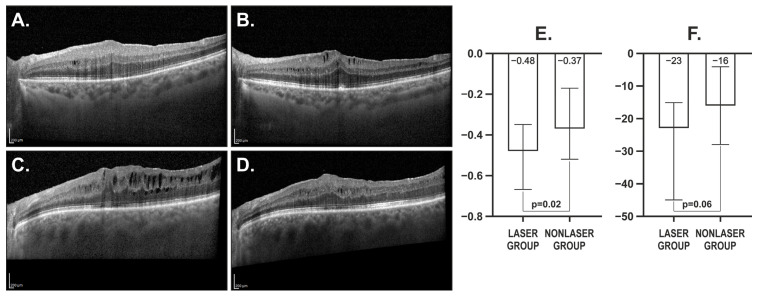
Retinal imaging obtained with Spectralis EDI-OCT and corresponding quantitative analysis. (**A**) Nonlaser control group, 1 month after pars plana vitrectomy (PPV); (**B**) Nonlaser control group, 4 months after PPV; (**C**) Laser group, 1 month post-PPV, before subthreshold micropulse laser therapy (SMLT); (**D**) Laser group, 4 months post-PPV, following SMLT. Scale bars, 200 μm; (**E**) Comparison of total retinal volume change (mm^3^) between laser and nonlaser groups; laser group: from 1 month post-PPV (pre-SMLT) to 4 months post-PPV (post-SMLT); nonlaser group: from 1 month post-PPV to 4 months post-PPV (without SMLT); (**F**) Comparison of central ETDRS retinal thickness change (μm) between laser and nonlaser groups; laser group: from 1 month post-PPV (pre-SMLT) to 4 months post-PPV (post-SMLT); nonlaser group: from 1 month post-PPV to 4 months post-PPV (without SMLT).

**Figure 2:**
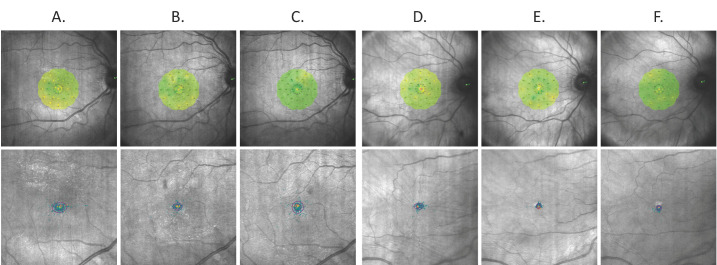
Representative macular sensitivity maps and fixation plots in patients from the nonlaser and laser groups before and after pars plana vitrectomy (PPV) and subthreshold micropulse laser treatment (SMLT). Columns A–C: nonlaser group—(**A**) before PPV, (**B**) 1 month after PPV, (**C**) 4 months after PPV. Columns D–F: laser group—(**D**) before PPV, (**E**) 1 month after PPV (prior to SMLT), (**F**) 4 months after PPV (3 months after SMLT).

We subsequently analysed the changes in BCVA, OCT, OCTA, microperimetry and electrophysiological parameters between the 1st and 4th months post-PPV. We found that the decrease in total retinal volume between the 1st and 4th months following PPV was significantly greater in the laser group than in the control group (median = −0.48, IQR = 0.32 vs. median = −0.37, IQR = 0.35; *p* = 0.02, respectively). Accordingly, the change in central ETDRS retinal thickness was greater in the SMLT group than in the nonlaser group, nearing statistical significance (median = −23, IQR = 30 vs. median = −16, IQR = 24; *p* = 0.06, respectively) (Figure 1). No differences were observed between the groups regarding BCVA changes or changes in any of the evaluated functional parameters (Figure 2).

### 3.3. The Relationships Between the Analysed Parameters

To characterize factors that may influence the change in total retinal volume in response to SMLT treatment, we evaluated the potential associations between the analysed parameters. In the laser-treated group, greater changes in total retinal volume were associated with more pronounced improvements in fixation stability parameters, showing moderate positive correlations for 63% BCEA: vertical (Rs = +0.41, *p* = 0.02), 63% BCEA: area (Rs = +0.40, *p* = 0.03), and 95% BCEA: vertical (Rs = +0.43, *p* = 0.02). Accordingly, larger reductions in total retinal volume were correlated with an increase in subfoveal choroidal thickness (Rs = −0.37, *p* = 0.03) and were associated with a more substantial reduction in the FAZ area in DVC (Rs = 0.38, *p* = 0.04). Interestingly, we also found correlations between changes in the FAZ area in SVC and fixation stability parameters, indicating that greater improvement in fixation stability was associated with larger change in the FAZ area in SVC, with moderate negative associations observed for 63% BCEA: horizontal (Rs = −0.49, *p* = 0.005) and 95% BCEA: horizontal (Rs = −0.47, *p* = 0.008).

Next, we aimed to describe the preoperative parameters that might improve the efficacy of SMLT treatment (Table 3). We found that the thicker the macula prior to PPV was, the greater the reduction in total retinal volume following SMLT was (Rs = −0.44, *p* = 0.008). For microperimetry characteristics, the lower the baseline macular sensitivity was, the greater the reduction in total retinal volume was (Rs = +0.66, *p* < 0.001). Similarly, the greater the degree of fixation stability within the area encompassing 95% of the fixation points was, the more pronounced the reduction in total retinal volume was (Rs = −0.34, *p* = 0.048). A lower preoperative *p*-wave amplitude in ring R1 on multifocal electroretinography, which reflected poorer initial subfoveal macular bioelectrical function, was also correlated with a greater reduction in total retinal volume (Rs = +0.39, *p* = 0.02). Moreover, the more advanced the baseline ERM stage was according to the Govetto classification and the lower the initial visual acuity was, the greater the decrease in total retinal volume post-SMLT (Rs = −0.46, *p* = 0.006; Rs = +0.35, *p* = 0.04, respectively). Notably, these correlations were not observed in the nonlaser-treated group.

## 4. Discussion

SMLT has emerged as a versatile and safe modality, with substantial evidence of efficacy in multiple non-surgical retinal diseases [12,13,14,15,16], and an expanding role in the management of post-surgical macular oedema, where favourable anatomical and functional outcomes have been reported.

Verdina et al. [17] reported that SMLT was a safe and effective option for the resolution of postoperative cystoid macular edema refractory to conventional therapies, occurring after cataract or retinal detachment surgery. The study included ten eyes with edema persisting for at least four months prior to SMLT treatment. Complete subfoveal macular edema resolution was achieved in all cases, with statistically significant improvements in BCVA and CMT (central macular thickness) at all follow-up visits, including at six months.

Bonfiglio et al. [18] conducted a prospective study on 95 eyes with persistent DME after PPV for tractional DME. SMLT treatment resulted in significantly lower CMT and better BCVA at 3 and 6 months compared to controls, supporting its role as a safe and effective option for post-PPV macular oedema.

Chen et al. [19] conducted a randomized controlled trial in 24 patients with persistent subretinal fluid for at least one month after scleral buckling surgery for rhegmatogenous retinal detachment, reporting a significant reduction in macular volume at 6 months in the SMLT-treated group compared to the observation group, with no adverse effects.

To date, only a very limited number of studies have explored the use of SMLT in patients who underwent ERM surgery. The potential role of SMLT in the postoperative management of the ERM was initially explored by Luttrull, who utilized a red (810 nm) diode laser, as opposed to the yellow (577 nm) laser used in our study. His findings demonstrated significant improvement in BCVA and maximum macular thickness (MMT), whereas central foveal thickness (CFT) remained unchanged. However, the interpretability of these results is limited by several methodological constraints, including a small sample size, retrospective design, lack of a control group, and absence of standardized follow-up intervals, all of which reduce the overall generalizability of the conclusions [2].

To the best of our knowledge, our study is the second to evaluate the effects of early postoperative SMLT following ERM removal in a prospective manner. In contrast to the previously published work of Lin et al. [11], our study included a larger patient cohort and employed more sophisticated tools to evaluate macular function, e.g., multifocal electroretinography and microperimetry. In our study, we observed that the decrease in total retinal volume between the first and fourth postoperative months was significantly greater in the SMLT group than in the control group. These findings are in line with the results reported by Lin et al., who observed a greater reduction in central subfield thickness (CST) in the SMLT group at the 3-month follow-up. However, neither our study nor the study of Lin et al. demonstrated the impact of SMLT on improvement in BCVA between the laser-treated and control groups. This might be due to the relatively short follow-up period, which limits the ability to observe long-term functional outcomes. A longer observation period, as well as repeated sessions of SMLT, could enhance the therapeutic effect and translate into more meaningful improvements in retinal function.

Notably, a reduction in total retinal volume after SMLT was associated with more pronounced improvements in fixation stability parameters in our study. This correlation suggests a potential link between the anatomical effects of SMLT and the functional enhancement of retinal performance.

In contrast to previously published data, we opted to initiate SMLT as early as possible, with a fixed time interval of one month after the operation. By this time, the intraocular sulfur hexafluoride (SF_6_) gas tamponade administered during surgery is typically fully resorbed, thereby eliminating any potential interference with laser delivery and ensuring optimal retinal visualization. We assumed that persistent accumulation of intraretinal fluid after the operation can further disrupt the retinal architecture, leading to photoreceptor damage and subsequent visual impairment [24]. Studies have demonstrated that prolonged macular thickening may further contribute to degeneration and functional impairment of the macula. As fluid accumulates in the macula, distortion of the retinal architecture occurs, leading to vision loss and, in chronic cases, potentially irreversible visual impairment due to scarring [9]. Thus, we cannot exclude the possibility that delayed SMLT application might have a lesser effect on macular morphology and function. In our study, the decision to administer SMLT at this early postoperative stage was driven by the intent to reduce macular oedema promptly, thereby promoting faster visual recovery and enhancing the overall surgical outcome.

Importantly, we also searched for potential mechanisms of action underlying SMLT. We performed correlation analysis to identify factors potentially contributing to changes in total retinal volume following SMLT. In the laser-treated group, a greater reduction in total retinal volume after SMLT was associated with a more pronounced decrease in the foveal avascular zone (FAZ) area within the deep vascular complex, as well as with an increase in subfoveal choroidal thickness. These findings suggest that the therapeutic effect of SMLT may be mediated, at least in part, by the modulation of vascular mechanisms. Indeed, a study by Viggiano et al. [25] demonstrated vascular effects of SMLT in patients with chronic central serous chorioretinopathy, with a significant increase in total choroidal thickness two months after treatment (*p* = 0.002). This observation supports the hypothesis that SMLT may influence the choroidal vasculature, potentially contributing to its therapeutic mechanism of action.

Accordingly, we demonstrated that the change in total retinal volume following SMLT was significantly correlated with changes in the deep vascular complex (DVC), which is consistent with previous reports by Vujosevic et al. [26] on patients with treatment-naive DME. These results may suggest that the therapeutic effects of SMLT are more closely linked to microvascular remodelling within the deeper retinal layers.

In our study, a greater reduction in total retinal volume three months after SMLT was also associated with more pronounced improvements in fixation stability parameters. This correlation suggests a potential link between the anatomical effects of SMLT and the functional enhancement of retinal performance.

Subsequently, we described the preoperative parameters that might affect the efficacy of SMLT. Our findings suggest that early postoperative SMLT may be particularly beneficial in patients presenting with more advanced preoperative retinal pathology. Greater reductions in total retinal volume were observed in eyes with increased preoperative macular thickness, lower baseline macular sensitivity, reduced fixation stability, and diminished subfoveal bioelectrical function. Additionally, higher ERM stage and poorer visual acuity prior to vitrectomy were also associated with a more pronounced anatomical response to SMLT. These correlations may help define the clinical profile of patients who are most likely to benefit from early postoperative SMLT following epiretinal membrane surgery. Indeed, Iuliano et al. [27] reported that patients with stage 4 idiopathic ERMs had an eightfold increased risk of developing persistent macular oedema within six months after surgery compared with those with stage 1–3 disease. These data support the rationale for implementing early interventional strategies such as SMLT in high-risk individuals, particularly those with advanced preoperative ERM features.

We acknowledge that the relatively short follow-up period (3 months after SMLT, corresponding to 4 months after PPV) represents a limitation of this study, as it may be insufficient to fully assess the effects of the SMLT treatment. Extending the follow-up to 6–12 months in future studies would allow a more comprehensive evaluation of the tested parameters, including both anatomical and functional outcomes.

## 5. Conclusions

Early postoperative application of SMLT has a favourable effect on retinal morphometric parameters by promoting the resolution of persistent macular oedema. However, this anatomical benefit does not result in a statistically significant improvement in functional macular outcomes. Nevertheless, SMLT may represent a viable adjunctive treatment strategy in select patients with advanced ERMs and poor baseline BCVA.

## Figures and Tables

**Table 1:** Baseline characteristics of the study groups.

Evaluated Parameters	SMLT Group	Control Non-Laser Group	*p* *
*n*	35	33	
Gender (F/M)	21/14	20/13	
Age Median (IQR) Mean ± SD	72 (9) 70.86 ± 6.03	72 (6) 71.97 ± 5.07	0.64
Govetto stage: 1—*n* (%) 2—*n* (%) 3—*n* (%) 4—*n* (%)	2 (6%) 10 (29%) 16 (46%) 7 (20%)	4 (12%) 10 (30%) 12 (36%) 7 (21%)	0.76
BCVA [Snellen charts] Median (IQR) Mean ± SD	0.4 (0.3) 0.44 ± 0.21	0.5 (0.2) 0.46 ± 0.2	0.75
BCVA [ETDRS charts] Median (IQR) Mean ± SD	22 (20) 19.29 ± 10.76	22 (8) 19.7 ± 9.39	0.91
Presence of metamorphopsia at the time of evaluation 1—Yes *n* (%) 0—No *n* (%)	26 (76%) 8 (24%)	22 (69%) 10 (31%)	0.58
Duration of metamorphopsia 1—<6 months *n* (%) 2—6–12 months *n* (%) 3—>12 months *n* (%)	9 (33%) 7 (26%) 11 (41%)	7 (30%) 8 (35%) 8 (35%)	0.79
Central ETDRS retinal thickness [μm] Median (IQR) Mean ± SD	497 (103) 502.11 ± 75.85	505 (121) 502.12 ± 91.12	0.93
Total retinal volume [mm^3^] Median (IQR) Mean ± SD	10.85 (1.35) 10.95 ± 1.15	11.12 (1.77) 10.97 ± 1.59	0.89
Choroidal area [mm^2^] Median (IQR) Mean ± SD	1.56 (1.9) 1.56 ± 0.42	1.54 (0.6) 1.6 ± 0.44	0.74
Subfoveal choroidal thickness [μm] Median (IQR) Mean ± SD	210 (117) 230.57 ± 79.04	245 (108) 247.97 ± 83.25	0.38
FAZ area in SVC [mm^2^] Median (IQR) Mean ± SD	0.13 (0.12) 0.16 ± 0.14	0.16 (0.18) 0.2 ± 0.18	0.3
FAZ area in DVC [mm^2^] Median (IQR) Mean ± SD	0.25 (0.29) 0.3 ± 0.24	0.24 (0.45) 0.39 ± 0.33	0.48
P1 wave amplitude in R1 [nV/degree^2^] Median (IQR) Mean ± SD	72.05 (47.27) 73.48 ± 26.05	63.43 (35.72) 67.43 ± 27.8	0.25
P1 wave implicit time in R1 [ms] Median (IQR) Mean ± SD	48 (4.9) 47.15 ± 4.07	45.1 (6.9) 46.61 ± 4.98	0.39
Average threshold [dB] Median (IQR) Mean ± SD	24.1 (3.7) 23.19 ± 3.36	24.5 (2.9) 24.37 ± 2.13	0.25
Fixation Stability P1 [%] Median (IQR) Mean ± SD	96 (13) 91.09 ± 11.71	98 (7) 94.12 ± 7.74	0.28
Fixation Stability P2 [%] Median (IQR) Mean ± SD	100 (2) 97.66 ± 5.38	100 (1) 98.42 ± 2.89	0.42
63% BCEA: area [deg^2^] Median (IQR) Mean ± SD	0.6 (1.5) 1.44 ± 2.45	0.4 (0.7) 2.7 ± 10.44	0.61
95% BCEA: area [deg^2^] Median (IQR) Mean ± SD	1.8 (4.5) 4.33 ± 7.32	1.3 (2.1) 8.11 ± 31.28	0.65

* Fisher exact test for categorical variables or Mann–Whitney U test for continuous and ordinal variables. IQR, interquartile range.

**Table 2:** Postoperative characteristics of the SMLT and control groups detected 1 month post-ERM surgery, before SMLT application.

Evaluated Parameters 1 Month After PPV	SMLT Group Median (IQR) Mean ± SD	Control Non-Laser Group Median (IQR) Mean ± SD	*p* *
BCVA [Snellen charts]	0.7 (0.4) 0.63 ± 0.21	0.6 (0.2) 0.61 ± 0.18	0.61
BCVA [ETDRS charts]	28 (14) 24.8 ± 9.83	28 (12) 26 ± 8.4	0.81
Central ETDRS retinal thickness [μm]	430 (57) 438.17 ± 39.64	443 (65) 448.1 ± 71.37	0.67
Total retinal volume [mm^3^]	9.78 (0.9) 9.83 ± 0.62	9.92 (1.03) 9.83 ± 0.93	0.65
Choroidal area [mm^2^]	1.51 (0.78) 1.55 ± 0.42	1.46 (0.47) 1.54 ± 0.36	0.95
Subfoveal choroidal thickness [μm]	228 (107) 234.46 ± 78.32	238 (89) 234.21 ± 68.49	0.9
FAZ area in SVC [mm^2^]	0.1 (0.08) 0.13 ± 0.11	0.1 (0.11) 0.14 ± 0.11	0.67
FAZ area in DVC [mm^2^]	0.17 (0.11) 0.18 ± 0.08	0.23 (0.17) 0.3 ± 0.29	0.07
P1 wave amplitude in R1 [nV/degree^2^]	75.45 (30.43) 73.86 ± 23.61	69.43 (20.15) 71.84 ± 24.28	0.59
P1 wave implicit time in R1 [ms]	50 (5.8) 49.57 ± 3.98	50 (5.9) 49.44 ± 4.67	0.88
Average threshold [dB]	24.3 (2.7) 23.62 ± 2.97	24.6 (3) 24.46 ± 2.17	0.43
Fixation Stability P1 [%]	98 (8) 94.22 ± 9.23	98 (4) 96.39 ± 6.66	0.32
Fixation Stability P2 [%]	100 (1) 98.44 ± 4.13	100 (0) 99.48 ± 1.55	0.35
63% BCEA: area [deg^2^]	0.4 (0.8) 0.94 ± 1.65	0.4 (0.4) 0.57 ± 0.78	0.39
95% BCEA: area [deg^2^]	1.2 (2.55) 2.83 ± 4.92	1.1 (1.2) 1.67 ± 2.33	0.35

* Mann–Whitney U test. IQR, interquartile range.

**Table 3:** Correlation between post-SMLT total retinal volume change and initial values of the analysed parameters before PPV. Statistically significant values are highlighted in bold.

Evaluated Parameters	Rs Before PPV	*p* *
BCVA [Snellen chart]	**+0.35**	**0.04**
BCVA [ETDRS charts]	+0.29	0.08
Govetto stage [1–4]	**−0.46**	**0.006**
Central ETDRS retinal thickness [μm]	**−0.44**	**0.008**
Choroidal area [mm^2^]	−0.27	0.12
Subfoveal choroidal thickness [μm]	−0.26	0.14
FAZ area in SVC [mm^2^]	+0.14	0.46
FAZ area in DVC [mm^2^]	−0.32	0.11
P1 wave amplitude in R1 [nV/degree^2^]	**+0.39**	**0.02**
P1 wave implicit time in R1 [ms]	+0.15	0.4
Average threshold [dB]	**+0.66**	**<0.001**
Fixation Stability P1 [%]	+0.24	0.17
Fixation Stability P2 [%]	+0.23	0.19
63% BCEA: vertical [°]	−0.33	0.051
95% BCEA: vertical [°]	**−0.34**	**0.048**
63% BCEA: area [deg^2^]	−0.29	0.09
95% BCEA: area [deg^2^]	−0.29	0.09

* Test for Spearman rank correlation coefficient.

## Data Availability

The data that were used to support the findings of this study are available from the corresponding author upon request.
